# Whole-body vibration training improves balance control and sit-to-stand performance among middle-aged and older adults: a pilot randomized controlled trial

**DOI:** 10.1186/s11556-017-0180-8

**Published:** 2017-07-18

**Authors:** Ming-Chen Ko, Long-Shan Wu, Sangwoo Lee, Chien-Chun Wang, Po-Fu Lee, Ching-Yu Tseng, Chien-Chang Ho

**Affiliations:** 1 Department of Physical Education, Fu Jen Catholic University, No. 510 Zhongzheng Road, Xinzhuang District, New Taipei City, 24205 Taiwan; 2 Department of Kinesiology, Texas Woman’s University, Denton, TX 762 USA; 3 Graduate Institute of Sports Training, University of Taipei, Taipei City, 11153 Taiwan; 4 Graduate Institute of Sport Coaching Science, Chinese Culture University, Taipei City, 11114 Taiwan

**Keywords:** Whole-body vibration training, Postural control, Balance, Limits of stability, Sit-to-stand test

## Abstract

**Background:**

Aging is associated with decreased balance, which increases falling risk. The objective of the current study was to determine the feasibility and effects of whole-body vibration (WBV) training on knee extensor muscle power, limits of stability, and sit-to-stand performance among community-dwelling middle-aged and older adults in the United States.

**Methods:**

A randomized pilot study with participant blinding was conducted. Feasibility outcomes included recruitment and compliance rate. Twenty-nine community-dwelling older adults were randomly assigned to perform body-weight exercises with either an individualized vibration frequency and amplitude, a fixed vibration frequency and amplitude, or no vibration. Isokinetic knee extensor power, limits of stability, and sit-to-stand tests were conducted before beginning the exercises (baseline) and after 8 weeks of training.

**Results:**

With a favorable recruitment rate (58%) and compliance rates (attrition 9%; adherence 85%), the intervention was deemed feasible. The limits of stability endpoint excursion score for the individualized frequency–amplitude group was increased by 8.8 (12.9%; *P* = 0.025) after training, and that group’s maximum excursion score was increased by 9.2 (11.5%; *P* = 0.006) after training. The average weight transfer time score was significantly decreased by 0.2 s in the fixed group. The participants in the individualized group demonstrated a significant increase (3.2%) in weight rising index score after 8 weeks of WBV training.

**Conclusions:**

WBV training is feasible for use with elderly people, and this study achieved good recruitment and compliance. The present paper suggests that 8 weeks of WBV training improves limits of stability and sit-to-stand performance. Future studies must determine whether WBV training improves other factors that affect posture control.

**Trial registration:**

This study was registered at the Texas Woman’s University Institutional Review Board [TWU IRB 17632] on the 3rd of November 2014.

## Background

One-third of community-dwelling individuals aged 65 years or older and approximately half of institutionalized people aged 80 years or older experience a fall each year [1]. Evidence also suggests that falling sideways from a standing position and landing on the trochanter region is strongly related to hip fracture [2]. Ten to 15 % of falls result in injury or head trauma [3]; although only approximately 1% of falls cause hip fractures, more than 90% of hip fractures are due to a fall [4]. Therefore, falls should not be considered random events or accidents. Impairment of muscle strength and power of the lower extremities, balance/postural control, and of the walking ability are known to be substantial risk factors for falls, and these parameters have been found to become progressively more impaired with aging [2, 5]. Therefore exercise should be generally accepted to effectively improve muscle strength, balance, and walking ability for the prevention of falls in older adults.

Whole-body vibration (WBV) training is a neuromuscular training modality that is used for strength training [6, 7]. In recent years, a systematic review and meta-analysis study conducted by Rogan et al. [8] reported that WBV has been introduced as a training method to improve muscle power and strength in older adults. Tonic vibration reflex (TVR) is the most commonly accepted theory explaining the beneficial effects of WBV training on exercise performance and balance [9]. The mechanical stimuli generated by vibration platforms are transmitted to the body and stimulate muscle spindles. This activates alpha motor neurons, which then causes reflexive muscle contractions [9, 10]. Increased muscle activity during WBV has been demonstrated using electromyography (EMG). For example, Hazell, Jakobi, and Kenno [11] reported that EMG activity was higher during the performance of dynamic semi-squats when WBV was involved than when it was not.

Delecluse et al. [6] compared the effects of WBV and resistance training on muscle strength, and found that 12 weeks of WBV (vertical sinusoidal vibration, 35–40 Hz; 2.5–5 mm peak-to-peak amplitude) significantly increased isometric and dynamic knee extensor torque. The magnitude of the strength increment was comparable to resistance training at moderate intensity (10–20 repetitions per set). Furthermore, only the participants in the WBV group achieved a significant increase in countermovement jump height. In addition to its beneficial effects on muscle strength, WBV has also been observed to improve balance. Torvinen et al. [7] reported that a single bout of WBV (four sets, 60 s per set) significantly increased knee extensor strength, vertical jump height, and postural control in young individuals. However, these beneficial effects subsided 60 min after WBV. Verschueren et al. [12] suggested that WBV training might also have positive effects on muscle strength and balance in postmenopausal women. After 6 months of WBV training, significantly increased isometric (16%) and dynamic (10.6%) knee extensor strength was discovered.

In another study, reduced anterior–posterior and mediolateral postural sway during arm abduction and anteflexion were used as indicators of improved postural control; the researchers determined that proprioceptive feedback from the ankle plays a crucial role in postural control [13]. Pollock et al. [14] reported that one session of WBV (five sets, 60 s per set) resulted in decreased cutaneous sensation. However, the results of balance tests did not reveal any significant changes. The explanation provided for this unchanged balance was that an insignificant tendency toward increased joint position sense might compensate for the decreased cutaneous sensation. Other studies have reported that after 8–12 weeks of WBV training, the knee movement detection threshold and joint position sense improved significantly in individuals with knee pathology [15, 16].

WBV training thus not only results in muscle function improvement, but also increases proprioceptor sensitivity. As previously discussed, resistance exercise training can improve muscle function and proprioceptive accuracy. However, elderly individuals were previously demonstrated to be at increased risk of falling or injury during strenuous load-bearing exercise [17]. Therefore, WBV training appears to be a promising alternative training modality. Unfortunately, the effects of this novel training modality on muscle function, balance, and proprioception, as determined from numerous studies, are inconsistent. This might be due to the different vibration frequencies, durations, and amplitudes used the studies. Furthermore, individual variability in the muscle damping coefficient might result in different people having distinct muscle responses even when following the same training protocol.

The objective of this pilot study was thus to use a randomized controlled trial to evaluate the feasibility and safety of using WBV training in the untrained elderly population and to determine the effects of body-weight exercise with an individualized WBV frequency on muscle function and balance. A fixed vibration frequency and no vibration were used as comparison treatments.

## Methods

The design of this study followed that proposed by Thabne et al. [18], which described how to report the results of a pilot study and also stated that the main purpose of a pilot study is to determine the feasibility of a larger study.

### Design

A randomized controlled pilot study was conducted that enrolled elderly participants who were randomly assigned into the following three groups: the individualized frequency–amplitude group, fixed-frequency group, and control group. Participants were blinded regarding their group assignment and a familiarization session was held before any test or intervention. During the familiarization sessions, the participants’ weight and height were obtained and the optimal vibration frequency and amplitude for each participant in the individualized frequency–amplitude group were determined. How the exercises should be performed on the vibration platform was also taught during the familiarization session. Baseline performance tests were conducted on the second visit of participants to the lab, and included limits of stability, sit-to-stand, and isokinetic knee extensor power tests. Post-intervention performance measurements (the same tests as baseline) were conducted 1 week after the 8-week WBV intervention.

### Participants

Participants were included if they fulfilled the following criteria: aged 55 years or above, able to stand with or without walking aids, and non-institutionalized. Individuals were excluded if they had a disease, were taking medications known to affect muscle strength, had had a recent fracture, had gall or kidney stones, had malignancies, were fitted with a cardiac pacemaker, or had already received WBV treatment.

### Randomization

Randomization was achieved by assigning a blinded research assistant to draw pieces of paper inscribed with participants’ names out of a box and divide the participants into the three groups (individualized frequency–amplitude, fixed-frequency, and control groups). The order of the measurements taken was also randomized for both the baseline and post-intervention tests. All participants gave written informed consent to the experimental procedure, which was approved by the Texas Woman’s University Institutional Review Board (TWU IRB 17632) and was in accordance with the Declaration of Helsinki.

### Protocol

The Power Plate pro5 (Power Plate North America Inc., U.S.A.) was used in this study. Its frequency can be adjusted from 25 to 50 Hz and its amplitude can be set at low or high (2 or 4 mm). Five vibration frequencies (30, 35, 40, 45, and 50 Hz) and two amplitudes (low and high) were utilized to determine the optimal frequency–amplitude combination for each participant in the individualized frequency–amplitude group. Participants were instructed to stand on the platform with their knees bent to an angle of 45°. The participants could grasp the rails attached to the platform as needed.

One week before the participants started any intervention, the optimal frequency and amplitude for each participant in the individualized frequency–amplitude group was determined by monitoring the electromyogram of the vastus lateralis and medial gastrocnemius muscles. The Noraxon Telemyo 900 (Noraxon Inc., U.S.A.) was the EMG system used to analyze muscle activity in this study, and the Myoresearch XP software (Noraxon Inc., U.S.A.) was employed to analyze the signals collected by the EMG system. The surface electrodes (Noraxon Inc., U.S.A.) were disposable, self-adhesive Ag–AgCl gel surface electrodes that had a diameter of 1 cm and an interelectrode distance of 2 cm. The skin over the muscle groups of interest was sterilized with alcohol pads and excessive hairs were shaven off if needed before the attachment of the electrodes, which were placed on the surface of the muscle. The EMG signals were amplified (1000×), band-pass filtered (10–500 Hz), rectified, smoothed (root-mean-square = 200 ms), and sampled at 1000 Hz (MyoResearch XP Masters Edition 1.04, Noraxon Inc., U.S.A.) before any analysis was performed. The patella was chosen as the reference site because no muscle activity is detected at the patella.

The participants were required to undergo WBV for 20 s in an isometric half-squat position under the following conditions: vibration at 0, 30, 35, 40, 45, and 50 Hz, tested in a random order. Each trial was separated by 2 min of rest. Two amplitudes were also tested to determine the optimal combination for each participant; therefore, each participant in the individualized frequency–amplitude group was required to perform 10 WBV trials. The average EMG signal (measured in microvolts; μV) during each 20 s trial was calculated, and the highest average EMG value obtained indicated the optimal intervention frequency and amplitude combination for each participant in the individualized frequency–amplitude group.

The participants in the two vibration groups underwent WBV three times a week for 8 weeks. There was a 24–48 h break between each vibration session (if the participant’s vibration training was on Monday, Wednesday, and Friday, the participant was asked to take two days off and resume the next cycle on Monday. If the participant’s vibration training schedule was on Tuesday, Thursday, and Saturday, the participant was asked to take two days off and resume the cycle on Tuesday). In each session, the participants performed five sets of WBV on the vibration platform, each set lasting 60 s. To ensure multidirectional, balanced vibration loading of the lower extremities, the following exercises were performed on the vibration platform: a light squat (knee angle 45°), standing in the full upright position, standing on tiptoe (30 s) and then switching to the heels (30 s), alternating the body weight from one leg to another (30 s for each leg), and back to a light squat. The participants were allowed to take a 1-min break between sets, during which they were required to sit on a chair. The vibration frequency and amplitude were set individually for each participant in the individualized frequency–amplitude group, whereas the vibration frequency was set to the average vibration frequency used for the individualized group for the participants in the fixed-frequency group. Participants in the control group did not perform any training.

### Primary outcome: Criteria of success

The criteria of success were based on the feasibility of the study protocol and focused on recruitment and compliance with the WBV training. The study was acceptable if one-third of the members of the LEAD-UP program at Texas Woman’s University eligible for the training were recruited, there was a 15% attrition rate, and there was an 80% training attendance rate. These recommended values were based on a previous pilot study [19]. To calculate the attrition rate, the number of participants who did not complete the final follow-up measurements was determined. To calculate the training attendance rate, the total number of training sessions completed by each participant was recorded. Each participant could participate in a total of 24 WBV training sessions. For safety reasons, the participants were also interviewed before and immediately after each WBV training session regarding whether they had experienced feelings of stability, well-being, dizziness, or pain during the vibration.

### Secondary outcome

The SMART Balance Master (Natus Medical Inc., U.S.A.) was used to measure the limits of stability (LOS) of the participants. The high validity and reliability values of measurement device and testing protocol have described and identified as being suitable for older adults [20]. This assessment determined their ability to voluntarily shift their center of gravity (COG) in eight directions in order to quantify the maximum distance they could travel without losing balance. The parameters that were measured were endpoint excursion (EPE), maximum excursion (MXE), and directional control (DCL). A sit-to-stand test was also performed on the SMART Balance Master. During the tests, the participants were required to be barefoot to eliminate the effects of different footwear. Foot alignment on the platform was determined according to each participant’s height and followed the instruction provided in the SMART Balance Master manual. A human shape cursor was displayed on a computer screen, which represented the participants’ COG. During the assessment, each participant was asked to move the cursor—using ankle strategies and weight-shifting only—to the highlighted targets. There were eight targets on the computer screen, positioned at 75% of the participants’ estimated LOS. The participants were required to reach the highlighted targets as fast as possible; the maximal time allowed to reach the targets was 8 s. Once the cursor reached the target, the participants were asked to hold that position until the trial was completed. The participants were told to get as close to the targets as they could without losing their balance or lifting their feet.

The following variables were used for the LOS test:

EPE: The distance traveled by shifting the COG to the highlighted targets; the endpoint was defined as the point at which the COG-shifting movement ceased.

MXE: The additional adjustments a participant makes to reach the targets.

DCL: The directional control represents whether the participants’ movement was directly toward the targets; a score of 100% DCL indicates that no path deviation occurred.

The composite scores for EPE, MXE, and DCL for each of the eight directions were used for further data analysis.

Isokinetic power tests were performed on the dominant leg using a Biodex Multi-Joint System 3 dynamometer (Biodex Medical Systems, Inc., U.S.A). The measurement device and testing protocol have described and identified as being valid and reliable in older adults [21]. The knee extensor muscle power was tested at 60° s^−1^ and 180° s^−1^ and the rotational axis of the dynamometer was aligned with the lateral femoral condyle of the dominant leg. The knee extension began at a joint angle of 90° and ended at 170°. The participants were required to perform one repetition of maximal knee extensor contraction at 60° s^−1^ and another repetition at 180° s^−1^. A 10-s break was taken between repetitions. The whole procedure was repeated once, and the two trials were separated by 5 min of rest. The average power was used for statistical analysis.

The sit-to-stand (STS) test determines a participant’s ability to transfer their COG from a seated to a standing position. The parameters measured were:

Weight transfer time (WTT): The time taken to transfer the COG forward, starting in a seated position and finishing with the complete body mass supported by the feet.

Weight rising index (WTRI): The total amount of force generated during the rising phase.

Center of gravity sway velocity (COGSV): The percentage of body mass carried by each leg during the rising phase.

The participants were required to be barefoot to eliminate the effects of different footwear. To perform the STS test, the participants were directed to sit on a box (the height of the box was 12 in) with arms placed by their sides. The participants were then requested to stand up as quickly as possible without any help from their arms or any other physical aid. The test was performed three times and the average scores were used for data analysis.

### Statistical analysis

All analyses were performed using IBM SPSS 19.0 (IBM Corp., Armonk, NY), and the significance level was set at *P* < 0.05. Statistical analysis was performed using a two-way (vibration groups × time) multivariate analysis of variance. The dependent variables were (a) EPE, MXE, and DCL for the LOS test, and (b) WTT, WTRI, and COGSV for the STS test. Because some of the data were skewed rather than normally distributed, differences between groups and tests were then determined by use of the Wilcoxon signed rank test and Friedman test. The effect sizes (ESs) were calculated and expressed as *r*. For *r*, an ES of 0.1 is considered a “small” effect, around 0.3 a “medium” effect and 0.5 and above, a “large” effect [22].

## Results

**Fig. 1:**
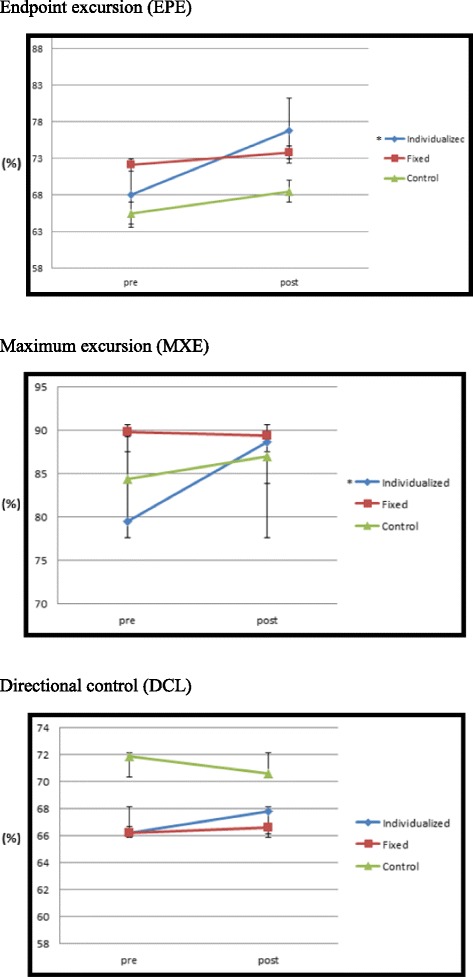
The endpoint excursion (EPE), maximum excursion (MXE), and directional control (DCL) scores for pre and post whole-body vibration (WBV) training. *Post-test values are significantly higher than pre-test values at *P* < 0.05

Figure 1 describes the flow of the participants through the study. All of them had been exercising at least two days per week for the preceding 6 months, and none were on any type of medication that would contaminate the data. The physiological characteristics of the participants are presented in Table 1.

**Table 1:** Physiological characteristics of participants

	Ind (*n* = 10)	Fix (*n* = 9)	Con (*n* = 10)
Age (years)	69.2 ± 7.0	65.7 ± 8.4	66.0 ± 4.1
Gender (Male/Female)	3/7	0/9	7/3
Height (cm)	167.4 ± 6.3	161.2 ± 2.3	174.5 ± 8.1
Weight (kg)	75.4 ± 9.8	65.1 ± 12.1	80.6 ± 12.8
BMI (kg/m^2^)	27.0 ± 4.2	25.0 ± 4.7	26.3 ± 2.5

All values are expressed as means ± SDs. *Con* control group, *Fix* fixed group, *Ind* individualized group, *SD* standard deviation

### Recruitment, attrition, and adherence

All of the participants were members of the LEAD-UP program at Texas Woman’s University. The program had a total of 115 members, of which staff representatives estimated 60 were still active. A total of 35 individuals volunteered to participate in the study during the recruitment process, resulting in a recruitment rate of approximately 58%. After they had completed a questionnaire during the screening interview, three participants were excluded because of the presence of an artificial cardiac pacemaker (*n* = 1), having a hip replacement that contained metal (*n* = 1), and having an inability to participate due to a scheduling conflict (*n* = 1). A total of 32 participants then signed the written informed consent, resulting in an inclusion rate-the proportion of participants invited to participate who enrolled-of approximately 91%. However, three participants dropped out before or after the first WBV training session for the following reasons: the use of a portable oxygen concentrator and the participant’s doctor suggesting no participation (*n* = 1); severe headache after the first WBV training session (*n* = 1); and cataract surgery (*n* = 1). A total of 29 participants completed all of the follow-up measurements (individualized frequency–amplitude group: *n* = 10; fixed group: *n* = 9; control group: *n* = 10), resulting in an approximately 9% attrition rate. The number of WBV sessions completed divided by the total number of training sessions offered indicated an excellent adherence rate of 85% to the study protocol over the 8-week training period. No side effects related to the intervention were reported.

### Secondary outcomes

#### Muscle power

The baseline and post-WBV-training isokinetic power measurements of each group are presented in Table 2. Notably, there was no significant between-group difference in knee extensor muscle power at 60° s^−1^ and 180° s^−1^ at baseline or after 8 weeks of training.

**Table 2:** Isokinetic power measurement

	Pre-test	Post-test	Change (%)	*P*	ES
**60 deg./s (Watts)**					
Ind (*n* = 10)	46.0 ± 17.5	50.1 ± 20.2	16.6 ± 43.5	0.252	0.588
Fix (*n* = 9)	45.8 ± 27.1	47.0 ± 23.1	13.6 ± 33.0	0.776	0.594
Con (*n* = 10)	66.0 ± 22.3	64.5 ± 24.1	−1.8 ± 17.6	0.606	0.600
**180 deg./s (Watts)**					
Ind (*n* = 10)	63.8 ± 26.6	73.5 ± 29.2	18.9 ± 27.2	0.077	0.589
Fix (*n* = 9)	67.6 ± 40.1	64.6 ± 44.3	−3.0 ± 28.0	0.670	0.594
Con (*n* = 10)	102.4 ± 50.9	93.6 ± 50.2	−9.3 ± 11.2	0.098	0.609

All values are expressed as means ± SDs. *Con* control group, *ES* effect size, *Fix* fixed group, *Ind* individualized group, *SD* standard deviation

#### Effects of WBV training on LOS

The baseline and posttraining results for EPE, MXE, and DCL are presented in Fig. 1. No baseline differences were observed in any of the LOS variables. EPE and MXE were significantly increased after 8 weeks of WBV training in the individualized frequency–amplitude group. The EPE for the individualized group at baseline was 68.0, which had increased by 8.8 (12.9%; *P* = 0.025) once the training had been completed. The MXE for the individualized group at baseline was 79.5, which had increased by 9.2 (11.5%; *P* = 0.006) once the training had been completed. No significant changes in EPE or MXE were observed in the fixed-frequency and control groups. Additionally, the baseline and posttraining results for DCL did not reveal significant differences for any group.

#### Effects of WBV on STS test

The baseline and post-WBV-training STS results for each group are presented in Table 3. After 8 weeks of WBV training, the average WTT had decreased by 0.2 s in the fixed-frequency group, and there was a significant increase (3.2%) in the WTRI among the participants in the individualized group. However, no significant change any group’s COGSV was noted.

**Table 3:** Sit-to-stand measurements

	Pre-test	Post-test	Change (%)	*P*	ES
**WTT (sec)**					
Ind (*n* = 10)	0.4 ± 0.2 (0.3)	0.4 ± 0.2 (0.3)	−2.9 ± 32.3	0.536	0.540
Fix (*n* = 9)	0.5 ± 0.3 (0.6)	0.3 ± 0.3 (0.3)	−36.0 ± 29.9	0.017*	0.674
Con (*n* = 10)	0.5 ± 0.2 (0.5)	0.4 ± 0.2 (0.4)	−7.4 ± 48.8	0.206	0.515
**WTRI (% of body weight)**					
Ind (*n* = 10)	20.8 ± 5.9	24.0 ± 6.0	17.8 ± 19.9	0.025*	0.580
Fix (*n* = 9)	22.4 ± 9.1	24.7 ± 9.4	13.8 ± 31.9	0.260	0.584
Con (*n* = 10)	24.7 ± 9.3	26.6 ± 9.5	11.0 ± 22.0	0.274	0.588
**COGSV (deg/s)**					
Ind (*n* = 10)	4.6 ± 1.8	4.7 ± 0.8	13.3 ± 37.7	0.868	0.573
Fix (*n* = 9)	3.4 ± 1.4	4.2 ± 1.1	34.5 ± 53.4	0.054	0.566
Con (*n* = 10)	3.8 ± 1.3	4.5 ± 0.9	30.7 ± 42.9	0.072	0.534

All values are expressed as means ± SDs (medians). *COGSV* center of gravity sway velocity, *Con* control group, *ES* effect size, *Fix* fixed group, *Ind* individualized group, *SD* standard deviation, *WTRI* rising index, *WTT* weight transfer time. *Post-test values are significantly different than pre-test values at *P* < 0.05

## Discussion

This randomized pilot study evaluated the feasibility of a WBV intervention in elderly people. In addition, the effect of 8 weeks of WBV training on muscle power, LOS, and STS performance was investigated.

The preliminary data provided useful information regarding the feasibility of WBV intervention for elderly people. Elderly individuals were successfully recruited, and no participant complained about and or reported side effects associated with the WBV training. This suggested that both individualized frequency–amplitude and fixed-frequency WBV training are feasible and safe intervention methods for the elderly population.

Improved postural control was also discovered in the current study. The data demonstrated that 8 weeks of WBV training using an individualized frequency and amplitude significantly improved participants’ EPE, MXE, and WTRI. A significant decrease in WTT was also discovered in the fixed-frequency group. Notably, this improved LOS and STS performance was not accompanied by any significant changes in knee extensor power.

Reduced postural control and increased risk of falling due to age have been reported by previous studies [23, 24], and lower limb muscle strength is one of the factors correlated with postural control [25]. Resistance training has been reported to improve lower limb strength and also postural control in elderly people [26, 27]. Improved balance after WBV training in individuals with anterior cruciate ligament injury and older individuals has also been reported [28–30]. However, the aforementioned studies only used a fixed vibration frequency and amplitude in their training. Thus, the effects of individualized vibration frequency and amplitude on muscle strength and postural control still remain unclear.

LOS has been described as the greatest distance an individual can intentionally move when shifting their COG toward a given direction without losing balance, stepping, or grasping [31]. As the distance an individual can shift their COG decreases, their base of support—which is used to maintain dynamic balance during any type of standing-based physical activity—weakens [32]. Accordingly, an individual’s LOS should be considered a critical prerequisite for the successful planning and execution of movements such as reaching forward to open a door or placing something on a shelf [33]. After 8 weeks of WBV training, only the participants in the individualized frequency–amplitude group demonstrated significant improvement in EPE and MXE. Bulat et al. [34] reported that 8 weeks of exercise training significantly improved EPE (from 40.9 ± 9.2 to 49.6 ± 11.4) and MXE (from 53.7 ± 10.3 to 63.9 ± 13.3). The training protocol adopted included lower extremity strengthening (stepping with an elastic band) and training for flexibility, coordination (dribbling soccer balls or completing an obstacle course), postural control (static standing with eyes closed), and gait (random, sudden changes in walking direction). The duration of each training period was 1 h (one session per week). The current study also identified improvement in EPE and MXE, obtained after 8 weeks of WBV training. The advantages of the WBV training protocol used in the present study are a shorter training duration per session (10 min) and a lower risk of falling during training sessions.

Several possible mechanisms may explain why individualized frequency–amplitude WBV training significantly improved LOS in the present study. For example, somatosensory stimulation (SSS) has been reported to trigger brain plasticity (modification of the maps in the brain cortex) [30, 35]. Van Nes et al. [30] indicated that WBV training (30 Hz, 3 mm peak-to-peak displacement, 45 s per set, four sets, 1 min pause between sets) significantly improved balance in individuals who had previously had a stroke. The authors suggested that WBV training was a strong SSS for both sides (paretic and unaffected) of the body that promoted brain plasticity and led to improved balance [30].

The improved LOS identified in the present study was supported by the study of Schuhfried et al. [29], which also used individualized WBV frequency. Those researchers began with a vibration frequency of 1 Hz and gradually increased it until each participant (individuals with multiple sclerosis) could not tolerate a further increase; the maximum frequency reached was then used for treatment. Significant improvements in postural sensory organization and timed get-up-and-go were reported. The stimulation of pressure receptors (Merkel nerve endings, Meissner’s corpuscles, Ruffini nerve endings), proprioceptors, and TVR are possible explanations for improved postural control [29, 36, 37]. However, caution is needed when interpreting the results of Schuhfried et al. [29] because the participants were only required to perform one bout of WBV exercise and the measurements were taken 15 min, 1 week, and 2 weeks after the WBV exercise. Johansson [35] proposed that repeated stimulation of skin receptors promotes brain plasticity and that the cortical representation of the muscles involved will remain enlarged. Therefore, it is possible that the 8 weeks of WBV training used in the current study repeatedly stimulated the skin receptors and muscle spindle, which might explain why improved LOS was discovered.

Fixed–frequency and amplitude WBV training has also been reported to significantly improve balance [38]. For example, Cheung et al. [39] demonstrated that 3 months of WBV training (20 Hz, 2 mm peak-to-peak displacement, 3 consecutive min on the vibration platform, three sessions per week) improved MXE (increased by 18.8% ± 18.3%) and DCL (increased by 4.3% ± 19.6%) in elderly women. Improved neuromuscular coordination may account for these improved MXE and DCL. In contrast with the study of Cheung et al. [39], the fixed–frequency and amplitude WBV training employed in the present study did not result in any meaningful changes in any of the balance variables. Identifying the exact mechanism that may account for why only participants in the individualized group exhibited balance improvements is not possible; however, a stronger SSS from the individualized vibration frequency and amplitude is a possible explanation because the vibration frequency and amplitude were individualized for each participant in the individualized group according to their EMG readings. Therefore, an individualized WBV protocol might enable the provision of a stronger stimulation to skin receptors, muscle spindles, and the vestibular system [29, 40].

Transferring the COG when moving from a sitting to a standing position is an essential daily movement and significant functional limitations can result if this ability is impaired [41]. STS tests have been used as a measurement for postural control, fall risk, lower limb strength, proprioception, and visual contrast sensitivity [42–44] for a variety of groups, such as individuals with arthritis, renal disease, stroke, and Parkinson disease, as well as older adults [45–48]. The transition from a sitting to a standing position changes the base of support from having three points to having two, which poses a challenge for dynamic stability and postural control [49]. A shorter STS time has been suggested to be an indication of higher postural and directional control [50]. Bhatt et al. [45] stated that the STS transition can be divided into two phases: movement preparation (the initiation of the movement; up until the lift-off) and execution (from the lift-off to the end of the movement). Individuals with Parkinson disease have lower hip flexion torques during the movement preparation phase and take longer to reach peak hip and knee extension torques during the execution phase, which leads to a longer completion time of the STS test [45].

Tung and Yang [51] reported that the significantly shorter STS time in individuals who had had a stroke after 4 weeks of STS training was due to higher hip extensor, knee extensor, and plantar flexor strength; similar results have also been observed after WBV training. Lee et al. [52] required participants (individuals with diabetic peripheral neuropathy) to perform WBV training (15–30 Hz, gradually increased from the first week to the sixth week; 2-mm peak-to-peak amplitude, three sets, 3 min per set) in addition to a balance exercise program (static and dynamic training). The results indicated that balance exercise training that included WBV resulted in significantly greater STS performance and postural control improvements compared with balance exercise training alone. Increased plantar flexor strength may have caused the improved performance. In the current study, 8 weeks of fixed-frequency WBV training significantly improved STS performance. Shorter WTT and higher WTRI after WBV training were indications of improved dynamic stability in the current study. Although one previous study [53] indicated that STS performance is related to lower limb muscle strength, concurrent improvement in knee extensor strength was not observed in the current study. Visual contrast sensitivity, lower limb proprioception, tactile sensitivity, and anxiety were also suggested as significant and independent predictors of STS performance [44]. Therefore, it is possible that the WBV protocol used in the current study improved factors related to STS performance other than muscle strength.

### Limitations

There are some limitations of the present study to be discussed. First, this study attempted to determine the most effective WBV-training frequency and amplitude combination for each individual to improve their knee extensor muscle power and postural control. However, the specific frequency and amplitude identified for each participant in the individualized group were determined according to EMG readings obtained during light squats (at a knee angle of 45°). It is possible that the optimal vibration frequency and amplitude combination might vary according to different leg positions. In further studies, the frequency and amplitude combination for different leg positions should be determined. Second, the knee extensor was the only muscle group measured. Whether WBV training can improve the strength of plantar flexors and hip extensors was not addressed in the present study. More muscle groups related to postural control and STS performance should be measured. Third, the participants in the present study were members of the LEAD-UP program and were already performing exercise at least two times per week. Hence, the intensity of the WBV training may not have been high enough to induce any significant change in knee extensor strength. To isolate the effectiveness of WBV training on the measured variables, untrained elderly individuals should be recruited.

## Conclusions

This pilot study demonstrated that the study protocol is feasible and safe for use with elderly individuals. Furthermore, the current study suggested that participants in the individualized frequency–amplitude group significantly improved their LOS and STS performance after 8 weeks of WBV training. Improved STS performance was also observed in the fixed-frequency group. However, 8 weeks of WBV training did not have any effect on knee extensor strength. Factors other than knee extensor strength—for example, improved hip extensor and plantar flexor strength, proprioception, and neuromuscular coordination—may account for the improved LOS and STS performance.

## Abbreviations

COG: Center of gravity
COGSV: Center of gravity sway velocity
DCL: Directional control
EMG: Electromyography
EPE: Endpoint excursion
ES: Effect size
LOS: Limits of stability
MANOVA: Multivariate analysis of variance
MXE: Maximum excursion
SD: Standard deviation
STS: Sit to stand
TVR: Tonic vibration reflex
WBV: Whole-body vibration
WTRI: Weight rising index
WTT: Weight transfer time
